# The prophylactic and therapeutic effects of moxibustion combined with traditional Chinese medicine decoction for treating chemotherapy-induced myelosuppression in early-stage breast cancer: study protocol for a randomized controlled trial

**DOI:** 10.1186/s13063-020-04749-6

**Published:** 2020-10-12

**Authors:** Yajie Ji, Siyu Li, Xinyue Zhang, Yu Liu, Qing Lu, Qiong Li, Weili Chen, Jiayu Sheng, Ke Jiang, Hongli Liang, Shanyan Sha, Mengting Li, Zongxin Chen, Peiyi Zheng, Minhong Wang, Yuanyuan Feng, Lei Wang, Huangan Wu, Huirong Liu, Yan Huang, Zhiguang Yin, Xiaohong Xue

**Affiliations:** 1 Department of Breast Surgery, Yueyang Hospital of Integrated Chinese and Western Medicine, Shanghai University of Traditional Chinese Medicine, Shanghai, 200437 China; 2 Shanghai Research Institute of Acupuncture and Meridian, Shanghai University of Traditional Chinese Medicine, Shanghai, 200030 China

**Keywords:** Breast cancer, Traditional Chinese medicine, Wenshen Shengbai decoction, Moxibustion, Chemotherapy-induced myelosuppression

## Abstract

**Background:**

Traditional Chinese medicine (TCM) has a long history of use in breast cancer, but lacking systematic evidence to support its clinical benefits. In this study, we evaluated the prophylactic and therapeutic effects of moxibustion combined with decoctions for treating chemotherapy-induced myelosuppression (CIM) in early-stage breast cancer patients.

**Methods:**

This is a randomized controlled clinical trial single-blinded for TCM decoction but not moxibustion. Patients are equally divided into the control group without decoction and moxibustion treatment (control), the decoction+moxibustion group (MD), and the placebo+moxibustion group (MP), according to the following stratification factors: age (below 40s, 40s, 50s, and 60s or above), chemotherapy regimen (anthracyclines, taxanes, anthracyclines+taxane, and others), and chemotherapy strategy (adjuvant and neoadjuvant). The TCM decoction is Wenshen Shengbai Decoction. The anticipated sample size is 462 cases (154 cases in each group). All participants are expected to treat with chemotherapy and recombinant human granulocyte colony-stimulating factor (rhG-CSF). The primary outcomes include the proportion of patients with relief of leukopenia and/or neutropenia, the myelosuppression-associated serious adverse event including grade 3–4 leukopenia and/or neutropenia, and febrile neutropenia, and the dose of rhG-CSF. The secondary outcomes include chemotherapy adherence, stratified analysis, adverse reactions, quality of life by EORTC Breast-Cancer-Specific Quality of Life Questionnaire including EORTC QLQ-C30 (V3.0) and QLQ-BR23, TCM Constitution, and 3-year disease-free survival and overall survival. Baseline information including age, surgical approach, chemotherapy regimen and strategy, pathological stage, and molecular subtype will be recorded.

**Discussion:**

This will be the first randomized controlled trial to evaluate the efficacy of moxibustion combined with TCM decoction in treating CIM in early-stage breast cancer patients, aiming to standardize the TCM decoction and moxibustion method, thus providing evidence for its clinical benefit.

**Trial registration:**

chictr.org.cn ChiCTR-INR-16009557. Registered on 23 October 2016.

## Administrative information

Note: the numbers in curly brackets in this protocol refer to SPIRIT checklist item numbers. The order of the items has been modified to group similar items (see http://www.equator-network.org/reporting-guidelines/spirit-2013-statement-defining-standard-protocol-items-for-clinical-trials/).

**Table:** 

Title{1}	The prophylactic and therapeutic effects of moxibustion combined with Traditional Chinese Medicine decoction for treating chemotherapy-induced myelosuppression in early-stage breast cancer: study protocol for a randomized controlled trial
Trial registration {2a and 2b}.	The study has been registered at chictr.org.cn (ChiCTR-INR-16009557) on 23rd October 2016.
Protocol version {3}	Protocol version: v2.1, Date: 28th February, 2018
Funding {4}	This work was supported by grants from the Three-year Programme of Shanghai Hospital Development Center (No. 16CR2026B) and National Natural Science Foundation of China (No. 81273642).
Author details {5a}	1. Department of Breast Surgery, Yueyang Hospital of Integrated Chinese and Western Medicine, Shanghai University of Traditional Chinese Medicine, Shanghai 200437, China 2. Shanghai Research Institute of Acupuncture and Meridian, Shanghai University of Traditional Chinese Medicine, Shanghai200030, China
Name and contact information for the trial sponsor {5b}	Yueyang Hospital of Integrated Chinese and Western Medicine, Shanghai University of Traditional Chinese Medicine (Address: 110 Ganhe Road, Hongkou District, Shanghai, China)
Role of sponsor {5c}	The funders did not involved in the study design, collection, management, analysis, and interpretation of data; writing of the report; and the decision to submit the report for publication.

## Introduction

### Background and rationale {6a}

Breast cancer is the most common malignant tumour in Chinese women, and its number of new cases and deaths accounts for 12.2% and 9.6% of the total globally [1]. Chemotherapy is one of the routine treatments for breast cancer [2–4]. For patients with early-stage breast cancer, dose-densed anthracycline combined with taxane regimens [5–7] or high-intensity chemotherapy [8, 9] is often recommended, yet such a combination regimen can cause acute cytotoxicity. While inhibiting tumour cell growth, it also kills haematopoietic cells and leads to significant myelosuppression, especially neutrophils [10]. Chemotherapy-induced myelosuppression (CIM) could lead to febrile neutropenia (FN), increase the risk of infection, and affect the survival of patients and thus usually results in the reduction in dose or even discontinuation of chemotherapy [11]. Therefore, the prevention and treatment of CIM are critical in breast cancer management.

At present, recombinant human granulocyte colony-stimulating factor (rhG-CSF) is commonly used in the prophylactic and therapeutic treatment of CIM [12–14]. rhG-CSF is a synthetic cytokine that promotes the proliferation, differentiation, and activation of neutrophils, mainly used for treating neutropenia after cytotoxic chemotherapy. rhG-CSF can significantly reduce the incidence of FN, shorten hospital stay, and reduce the use of intravenous antibiotics without affecting the efficacy of anti-tumour therapy [15, 16]. Prophylactic use of rhG-CSF is considered in patients with dosing reduction or delayed chemotherapy which may lead to an unfavourable prognosis [17–19]. Although the short-term effect of rhG-CSF is prominent, its rapid white blood cell boosting effect does not last long. Also, it may cause side effects such as bone pain, fever, spleen rupture, myelodysplastic syndrome (MDS), and acute myeloid leukaemia (AML) [20–23], which seriously upset the quality-of-life (QoL) and life expectancy of patients.

Nowadays, the value of traditional Chinese medicine (TCM) and moxibustion as a comprehensive treatment approach in breast cancer is getting well-recognized [24]. A large number of clinical observations and experimental studies have manifested that TCM decoctions can reduce surgical complication rate, promote postoperative physical recovery, attenuate chemotherapy toxicity, diminish the risk of recurrence and metastasis, and reverse drug resistance. Moxibustion has also demonstrated beneficial effects in preventing CIM in cancers [25]. However, the application of TCM decoctions and moxibustion on preventing and treating CIM in breast cancer remains controversy. First, there are a limited number of studies exploring the optimal TCM regimen and dose for breast cancer. Second, various means of TCM regimens are available, leading to a lack of consistency between studies. Third, the moxibustion method is not standardized. Last but not the least, there is a lack of randomized controlled trials to support the efficacy of moxibustion combined with TCM decoction in treating breast cancer patients.

## Objectives {7}

The present study aims to evaluate the efficacy of moxibustion combined with TCM decoction in treating breast cancer patients with CIM, in a randomized controlled setting. Through this study, we intend to establish a standardized TCM regimen and moxibustion method and provide evidence for the clinical benefits of moxibustion combined with TCM decoction, in compliance with current clinical practice guidelines.

## Trial design {8}

**Fig. 1:**
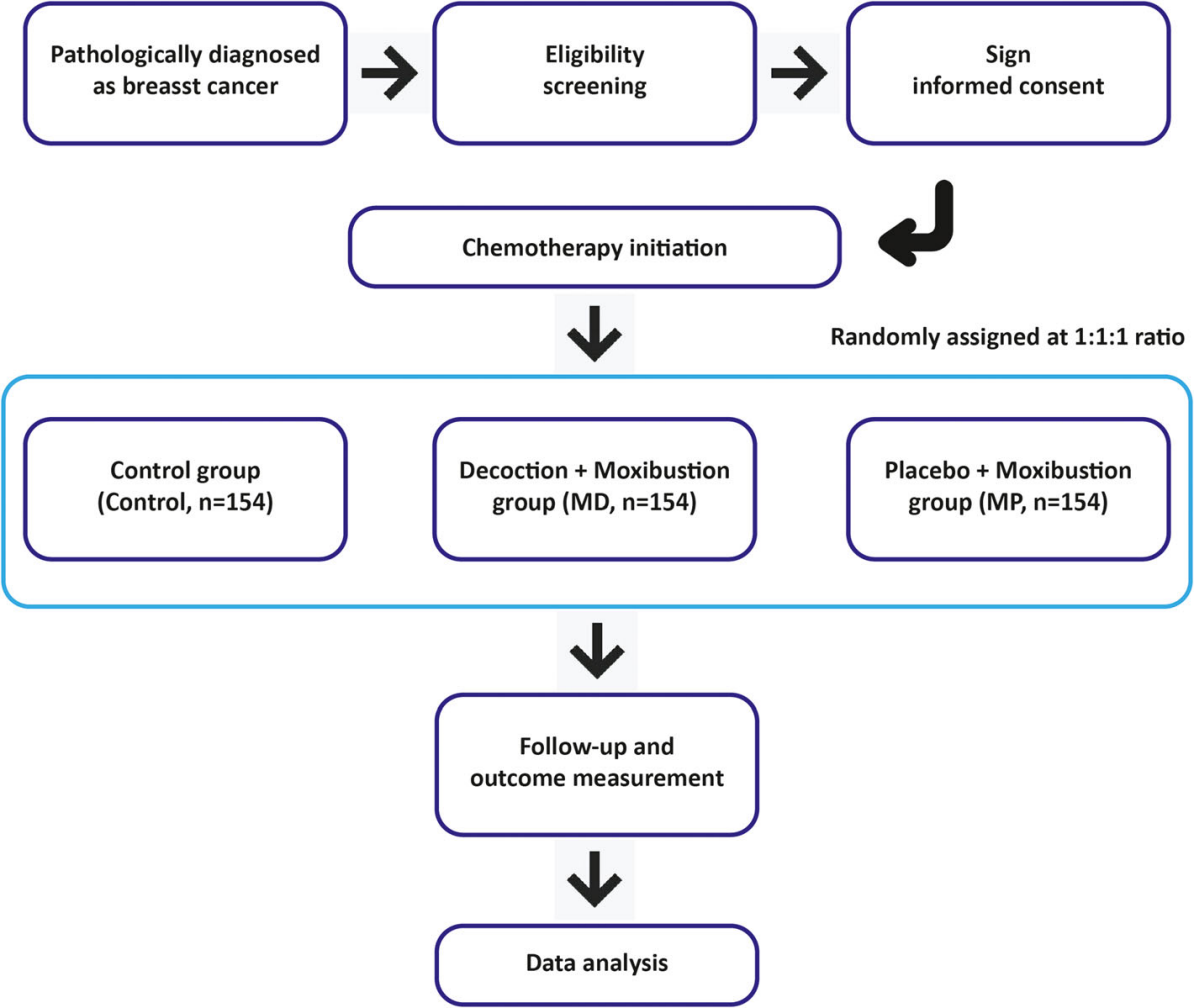
Study flow

This study is a randomized controlled clinical trial single-blinded for TCM decoction but not moxibustion. Patients will be divided into groups by stratified randomized grouping. The stratification factors involve age (below 40s, 40s, 50s, 60s, or above), chemotherapy regimen (anthracyclines, taxanes, anthracyclines combined with taxane, and others), and chemotherapy strategy (adjuvant and neoadjuvant). All eligible patients are equally divided into the control group with no TCM decoction/moxibustion treatment (control), the TCM decoction combined with moxibustion group (MD), and the placebo combined with moxibustion group (MP). All participants are expected to treat with standard chemotherapy and rhG-CSF regimen. The detailed study flow is illustrated in Fig. 1. The clinical pathway of each participant is shown in supplementary figure 1. Patients or the public will not be involved in the design, or conduct, or reporting, or dissemination plans of our research.

## Methods: participants, interventions, and outcomes

### Study setting {9}

Patients will be recruited from Yueyang Hospital of Integrated Chinese and Western Medicine, Shanghai University of Traditional Chinese Medicine.

### Eligibility criteria {10}

The inclusion criteria were as follows:

1. Patients should meet both the TCM and the western diagnostic criteria. A pathological diagnosis is required for every recruited patient.
2. Patients must be female.
3. Aged 18 to 80 years old.
4. Pathologically confirmed as primary invasive breast cancer.
5. Expected to undergo chemotherapy according to the guidelines.
6. Patients who agreed to, and are expected to, receive and complete a full course of chemotherapy.
7. White blood cell (WBC) count and absolute neutrophil count (ANC) before chemotherapy must be higher than the upper limit of the normal value.
8. Patients with good compliance in terms of language and willingness to follow-up.
9. Signed written informed consent obtained.

For inclusion criteria 1, the clinical diagnosis is in accordance with the “Tumor of the Breast (Second Edition)” edited by Shao Zhimin. The pathological diagnosis is in accordance with the “WHO Classification of Tumours of the Breast (4th Edition, 2012)”. The diagnosis and staging are based on clinical examination and post-operative pathological results according to the cancer staging criteria described in American Cancer Joint Committee (AJCC) (7th Edition). For the TCM diagnostic standard, the diagnosis is in accordance with the “SHIYONG ZHONGYI WAIKEXUE (2nd Edition, June 2010)” edited by Lu Deming and “Guideline for Diagnosis and Treatment of Tumour in TCM (ZYYXH/T135-156-2008)” edited by the China Association of Chinese Medicine. Chemotherapy regimen is based on CACA Guidelines for Breast Cancer Diagnosis and Treatment (2015 Edition) and NCCN Clinical Practice Guidelines in Oncology: Breast Cancer (Version 3. 2015).

The exclusion criteria were as follows:

1. Prior history of receiving systemic chemotherapy or radiotherapy.
2. Severe liver and kidney dysfunctions (serum ALT or AST is > 2.5 times the upper limit of the normal value and serum Cr is ≥ 150 μmol/L).
3. Severe heart dysfunction or other organ failures.
4. Any previous or concurrent primary malignancy.
5. Pregnant (serum β-HCG test) or lactating women.
6. Participating in other clinical trials 3 months prior to enrolment.
7. Cannot participant in the study due to any reasons such as language issues, geometrically unable to follow up, etc.
8. Other conditions or diseases that may result in significant risk to the patients as determined by the investigator.

### Who will take informed consent? {26a}

Written, informed consent to participate will be obtained from all participants through their responsible physicians. Participating in the study is voluntary. Participants may withdraw at any time without any given reason and the course of treatment will not be affected.

### Additional consent provisions for collection and use of participant data and biological specimens {26b}

On the consent form, participants will be asked if they agree to use their data in our ancillary study. Participants will also be asked for permission for the research team to share relevant data with people from the university taking part in the research or from regulatory authorities, where relevant.

## Interventions

### Explanation for the choice of comparators {6b}

The control group is treated routinely without either TCM decoction or moxibustion. Both the MD and MP groups are treated with moxibustion. The MD group is additionally treated with TCM decoction, while TCM placebo is provided to the patients in the MP group.

The TCM placebo which is made of excipients and flavouring agents is used as a comparator. The packaging, shape, weight, odour, and colour of the placebo are identical to that of the Wenshen Shengbai Decoction. The placebo is manufactured by Jiangyin Tianjiang Pharmaceutical Co., Ltd., and is stored and provided to the patients by the Central Pharmacy of Yueyang Hospital.

### Intervention description {11a}

#### Moxibustion

##### Acupoints

Shenque (CV8), Qihai (CV6), Guanyuan (CV4), bilateral Zusanli (ST36), and bilateral Sanyinjiao (SP 6) acupoints were selected. The rationale for moxibustion acupoint selection, treatment course, and duration has been described in a previous review published by our team [25]. Shenque (CV8), Qihai (CV6), and Guanyuan (CV4) belong to Ren meridian and govern the essence, blood, fluid, and fluid of the whole body.

##### Justification of acupoints

In the Inner Canon of Huangdi, the relationship between breast and meridian was described. The Stomach Meridian of Foot-Yanming runs downward along the midclavicular line and cross Ruzhong. The Spleen Meridian of Foot-Taiyin arises from the stomach, ascends through the diaphragm, and enters the chest. The Liver Meridian of Foot-Jueyin ascends through the diaphragm, distributes in the costal and the hypochondriac regions, and curves around the nipple. The Kidney Meridian of Foot-Shaoyin runs into chest to meet with breast. The Ren meridian ascends along the anterior medial line to the Guanyuan and enters the chest. The Chong meridian runs along both sides of the umbilicus and spreads over the chest. Therefore, current doctors generally accept that women’s nipple belongs to liver, and breast belongs to stomach. Thus, breast disease has a close relationship with zang-fu of liver, kidney, spleen, and stomach, as well as meridians of Chong-Ren. Zusanli belongs to the Stomach Meridian of Foot-Yanming and plays a role in assisting digestion, regulating spleen and stomach, reinforcing weakness, and regulating Qi and blood. Sanyinjiao originating from the postnatal essence belongs to the Spleen Meridian of Foot-Taiyin. It is the main intercept of the liver, spleen, and kidney meridians, which can strengthen the spleen and blood and regulate the liver and kidney [26]. Shenque, Qihai, and Guanyuan belong to the Ren meridian originating from the uterus, supervising the essence, blood, and body fluids from the whole body. Guanyuan is the chamber for essence and blood. Moxibustion of the Guanyuan acupoint can invigorate the kidney and Yang, regulate the Chong and Ren meridians, and harmonize Qi and blood [26]. Qihai is closely associated with the Qi of lung and is a key to the body’s Qi mechanism. Moxibustion of the Qihai can regulate the Qi mechanism and improve body health. Shenque is a key acupoint connecting to the whole body. It warms up the original Yang and runs the Yang-qi from the whole body. Therefore, the selection of the above acupoints considered to reinforce both the inborn and acquired conditions, aiming to warm up the kidney and Yang, adjust the essence, and nourish the blood.

##### Methodology

Fully expose the acupoints with mild moxibustion method, that is, ignite the moxa sticks, and then apply them to the acupoints. Cut a 1.5-cm-long moxa stick (ensure that the size and density meet the required specifications). The ignited moxa sticks should remain about 2–3 cm away from the skin to allow the patient to feel warm, but not burning (local skin should not appear red). The patient should be lying down, and the three acupoints including Shenque, Qihai, and Guanyuan should simultaneously apply moxibustion. Next, the patient should be sitting down, and Zusanli (both sides) followed by Sanyinjiao (both sides) should apply moxibustion. Each moxibustion lasts for 15 min. Gently massage the acupoints after moxibustion. The moxibustion should be performed once daily.

##### Course of treatment

Moxibustion starts from the 2nd day after chemotherapy and continued daily moxibustion until the day before the next cycle of chemotherapy. No moxibustion was given on the day of drug administration. The treatment course is completed 3 weeks after the end of the last chemotherapy. Daily self-moxibustion is carried out by the patient at home and is guided by Yueyang Hospital and Shanghai Acupuncture and Meridian Research Institute. Shanghai Research Institute of Acupuncture and Meridian will provide technical support for moxibustion.

#### TCM decoction

##### Prescriptions

Wenshen Shengbai Decoction consists of *Astragalus*, *Codonopsis pilosula*, *Atractylodes macrocephala*, *Poria cocos*, *Adenophora tetraphylla*, *Lycium chinense,* *Epimedium*, *Cistanche deserticola*, *Morinda officinalis*, *Curcuma zedoaria*, *Salvia chinensis*, *Sophora flavescens*, *Rhizoma polygonati*, *Cornu cervi*, and *Psoralea corylifolia* [27]. The herbs are boiled and extracted into granules. It is orally administrated daily from the seventh day of each cycle of chemotherapy to the first day before the next cycle of chemotherapy, aiming to avoid the acute gastrointestinal reaction. The same amount of granules is put into the same container with 50-mL warm water. Stir until the particles are completely dissolved, and then add an appropriate amount of water to dilute. The granule is made by Jiangyin Tianjiang Pharmaceutical Co., Ltd., and is stored and provided to the patients by the Central Pharmacy of Yueyang Hospital.

##### Rationale of TCM decoction

Post-operative CIM in breast cancer often causes fatigue, dizziness, and unwillingness to speak, palpitations, insomnia and dreaminess, spontaneous sweating and tachypnea, pale and enlarged tongue, weak pulses, etc. Although the concept of myelosuppression was not mentioned in ancient TCM, these clinical symptoms could be classified as a kind of “blood deficiency” and “consumptive diseases”. The treatment of CIM should, therefore, base on the concept of “tonifying the deficiencies” and “warming the consumptions”.

The *Astragalus*, *Codonopsis pilosula*, *Atractylodes macrocephala*, and *Poria cocos* in Wenshen Shengbai Decoction can tonify Spleen and support Qi and secure the postnatal condition, thus activating Qi and developing blood; *Adenophora tetraphylla* and *Lycium chinense* can nourish Yin and promote body fluid; *Epimedium, Cistanche deserticola, and Morinda officinalis* can tonify the kidney, regulate Chong Ren, and constrain the prenatal condition, thus enhancing the healthy Qi and resistance to pathogenic factors; *Curcuma zedoaria* and *Salvia chinensis* can promote blood circulation and detoxification and resolve phlegm; *Sophora flavescens* can clear away heat and dampness; *Rhizoma polygonati*, *Cornu cervi*, and *Psoralea corylifolia* can tonify kidney and fill the essence.

#### rhG-CSF

The rhG-CSF is performed according to the guidelines [12–14]. Patients with grade 1 myelosuppression according to the CTCAE 4.0.2 criteria are follow-up for both the intervention group and the control group. Patients with grade 2 or above myelosuppression should receive rhG-CSF treatment until the WBC count and ANC return normal. Two types of adjuvant chemotherapy regimens in breast cancer, namely ddAC-wP and TAC, can lead to a high risk of FN (incidence > 20%). According to the NCCN guidelines, prophylactic rhG-CSF treatment is recommended on day 2 or days 3 to 4 after chemotherapy. In detail, 5 mcg/kg is administrated per day until neutrophil count returns normal or nearly normal level, or long-acting rhG-CSF (PEG-rhG-CSF) is given on the second day after chemotherapy.

#### Chemotherapy regimen

The investigator is responsible to decide whether adjuvant chemotherapy or neoadjuvant chemotherapy is suitable for the patients according to the CACA Guidelines for Breast Cancer Diagnosis and Treatment (2015 Edition) and NCCN Clinical Practice Guidelines in Oncology: Breast Cancer (Version 3. 2015). Choices of chemotherapy regimens include anthracycline (EC × 4 times, CEF × 6 times), taxanes (TC × 4–6 times, P × 4–6 times), anthracycline combined taxanes (TAC × 6 times, FEC × 3 followed by T × 3 times, AC × 4 followed by T × 4 times, AC × 4 followed by wP × 12 times, ddAC × 4 followed by wP × 12 times), and others (PC × 4–6 times, DP × 4–6 times, NX × 4–6 times, X × 4–6 times).

### Criteria for discontinuing or modifying allocated interventions {11b}

Participants who show any adverse effects will be reported and followed up by their responsible physicians. A reduction in dose or discontinuation of treatment will be decided by the physician according to the CACA Guidelines for Breast Cancer Diagnosis and Treatment (2015 Edition) and NCCN Clinical Practice Guidelines in Oncology: Breast Cancer (Version 3. 2015), or upon participant request. Outcome data will be collected as usual according to the protocol for these participants.

### Strategies to improve adherence to interventions {11c}

To improve the adherence and promote participant retention, the required amount of moxa sticks and TCM granule will be provided by the Central Pharmacy of Yueyang Hospital to the corresponding participants each time after receiving chemotherapy, for free. For participants who decide to discontinue the treatment and withdraw from the study, they will be asked to fill a questionnaire to simply explain the reason behind. After discontinuation, outcome data will not be collected for these participants.

### Relevant concomitant care permitted or prohibited during the trial {11d}

No specific concomitant care and interventions are permitted or prohibited during the trial.

### Provisions for post-trial care {30}

None.

### Outcomes {12}

#### Primary outcomes

Primary outcomes are as follows:

1. The proportion of patients with relief of leukopenia and/or neutropenia.
2. The number and proportion of patients with relief of myelosuppression-associated SAE, defined as grade 3–4 leukopenia and/or neutropenia and FN.
3. The median and mean duration time of relief of leukopenia and/or neutropenia.
4. The dosing of rhG-CSF.

The grading of adverse reactions is in accordance with the CTCAE 4.0.2 criteria. Leukopenia is defined as an abnormal decrease in white blood cells of less than LLN-3.0 × 10^9^/L. Neutropenia is defined as an abnormal decrease in netrophils of less than LLN-1.5 × 10^9^/L. The definition of FN is based on the IDSA guidelines (American Society of Infectious Diseases Guide 2010 edition), which is an absolute neutrophil count (ANC) of < 500 neutrophils/mcL or an anticipated decline to ≤ 500 in the next 48 h and progress to ≥ 38.3 °C orally or ≥ 38.0 °C for a duration over 1 h.

#### Secondary outcomes

Secondary outcomes are as follows:

1. Chemotherapy adherence: The percentage of patients who completed the full-course chemotherapy in a standard dose. If only the full course of treatment is completed but the dose of chemotherapy is reduced, or the dose is unchanged but the course of treatment is shortened, the case will be treated as poor adherence.
2. Stratified analysis: Subgroup analysis will be done according to age, chemotherapy regimen, and chemotherapy strategy.
3. Adverse events: Any toxic side effects such as general condition, gastrointestinal reactions, liver and kidney function, skin reactions, burns, and heat syndrome will be recorded.
4. ECOG PS: The performance status of the patients before and after the completion of the whole treatment cycle according to the Eastern Cooperative Oncology Group.
5. Quality-of-life survey: EORTC QLQ-C30 (V3.0) and EORTC QLQ-BR23 will be used to assess the QoL of patients. QLQ-C30 is a scale developed by the European Organization for Research and Treatment of Cancer (EORTC) to measure the QoL of cancer patients. QLQ-BR23 is a QoL questionnaire designed for breast cancer patients, which is suitable for evaluating all breast cancer patients regardless of the type of treatments. Also, a TCM Constitution survey based on the judgement criteria recognized by the China Association of Chinese Medicine will be conducted [28]. The TCM Constitution is assessed by filling out the “the Criteria of Classification and Judgment of TCM Constitution (ZYYXH/T157-2009)” (Table 1). Every patient is designed to respond to the questionnaire three times: (1) before chemotherapy initiation, (2) during mid-term chemotherapy, and (3) before the last cycle of chemotherapy. All assessments will be completed the day before chemotherapy administration.
6. Survival: The differences in 3-year DFS and OS between the treatment group and the control group will be analysed.

**Table 1:** “The Criteria of Classification and Judgment of TCM Constitution” scale

Classification	Conditions	Judgement
Normal constitution	A conversion score of ≥ 60, and conversion score of < 30 in all other 8 types	Yes
	A conversion score of ≥ 60, and conversion score of < 40 in all other 8 types	Generally yes
	Unsatisfied for the above conditions	No
Abnormal constitution	A conversion score of ≥ 40	Yes
	A conversion score of 30 to 39	Likely yes
	A conversion score of< 30	No

The scale consists of 9 types including constitution of Gentleness (type A), Qi-deficiency (type B), Yang-deficiency (type C), Yin-deficiency (type D), Phlegm-wetness (type E), Wetness-heat (type F), Blood-stasis (G type), Qi-depression (H type), and Special diathesis (type I). Each type is graded in 5 contents. The original score is the sum of all items. Conversion score = [(original score − number of items)/(number of items × 4)] × 100

### Participant timeline {13}

The time schedule of enrolment, interventions, and assessments is as shown in Table 2.

**Table 2:** TCM-related symptom assessment table

Symptoms	No effect	Partial improvement	Significant improvement
Abdominal distension or pain	0	1	2
Abnormal leucorrhea	0	1	2
Blurred vision	0	1	2
Chest tightness or pain	0	1	2
Chills	0	1	2
Constipation	0	1	2
Diarrhoea	0	1	2
Dizziness and tinnitus	0	1	2
Dreaminess	0	1	2
Dry bitter mouth	0	1	2
Dry eyes	0	1	2
Dysuria	0	1	2
Easy to catch a cold	0	1	2
Epigastric discomfort	0	1	2
Fatigue	0	1	2
Fingers and toes numb	0	1	2
Hair loss	0	1	2
Headache	0	1	2
Hectic fever	0	1	2
Heel pain	0	1	2
Insomnia	0	1	2
Irregular menstruation	0	1	2
Irritability	0	1	2
Itchy or sore throats	0	1	2
Loss of appetite	0	1	2
Lower limb pain	0	1	2
Memory loss	0	1	2
Nausea	0	1	2
Palpitations	0	1	2
Perspiration	0	1	2
Sticky mouth	0	1	2
Tasteless	0	1	2
Throat obstruction	0	1	2
Waist or knee pain	0	1	2

The efficacy is assessed on a scale of 3 levels. No improvement: No change in integral value after treatment or the integral value after treatment decreased by < 30% compare to that before treatment. Partial improvement: The integral value after treatment decreased by ≥ 30% compare to that before treatment. Significant improvement: The integral value after treatment decreased by ≥ 70% compare to that before treatment

### Sample size {14}

In previous studies, the incidence rate of myelosuppression was 81.0% in the control group and 61.7% in the TCM decoction group [29]. The cure rate and effective rate of the moxibustion group were 84.1% and 66.4%, while those of the control group were 35.2% and 33.3% [30]. In our preliminary study, the bone marrow suppression rate (grade 2 or above) was 52% and 81% for the MD group and the control group, respectively (δ = 15%in superiority trial). Since there are no previous data supporting the combination use of moxibustion and TCM decoction, the sample size calculation was simply based on MD/MP group versus control group, assuming that the effect of combination treatment is equivalent to or better than moxibustion or decoction alone. Considering patient enrolment at a ratio of 1:1:1 for the control, MD, and MP groups, the sample size is calculated by multiple sample rate comparisons. The sample content estimation formula is as previously described [31]. Given that *α* = 0.05 and 1-*β* = 0.08, taking a 20% drop rate into account, the required total sample size is 462 cases and 154 cases in each group.

### Recruitment {15}

None.

## Assignment of interventions: allocation

### Sequence generation {16a}

The administrative staffs are responsible for generating the random allocation sequence using SPSS and providing the treatment allocation to the physicians in sealed envelopes.

### Concealment mechanism {16b}

As mentioned above in {16a}.

### Implementation {16c}

The physicians are responsible for patient enrollment and the assignment of participants to corresponding interventions according to the treatment allocation.

## Assignment of interventions: blinding

### Who will be blinded {17a}

Participants in the MD and MP groups are blinded to the use of TCM. The required amount of TCM granule or TCM placebo will be provided to the corresponding participants according to the grouping each time after chemotherapy. In consideration of the patient’s safety, outcome assessments are not blinded to the physicians.

### Procedure for unblinding if needed {17b}

None.

## Data collection and management

### Plans for assessment and collection of outcomes {18a}

Paper-based case report forms are filled by the physicians and are securely delivered to the trial office by each corresponding physician as soon as possible. The filled data is double-checked by administrative staffs who are responsible for data entry and maintenance of the electronic database. Data will only be accessible to the physicians responsible for the data analysis after the completion of patient recruitment. The template of case report form is available upon request.

#### Baseline information

Baseline information including age (below 40s, 40–60s, 60s, or above), surgical approach (breast-conserving and axillary conserving), chemotherapy regimen (anthracyclines, taxanes, anthracyclines + taxanes, and others), chemotherapy strategy (adjuvant and neoadjuvant), pathological staging (stages I, II, III), and molecular subtype (Luminal-like, HER-2 positive, and Basal-like) will be recorded.

#### Short-term efficacy

1. Blood routine test: Monitoring WBC count and ANC once a week during every cycle of chemotherapy.
2. TCM-related symptom: The investigators will evaluate the TCM-related symptoms based on the scoring method (Table 3) described in “Chinese Medicine New Drug Clinical Research Guidelines (Trial)” (China Medical Science and Technology Press, 2002) every 3 months.
3. Adverse effects: Grading on a scale of 0 to 4 according to CTCAE 4.0.2.
4. Quality-of-life: EORTC QLQ-C30, QLQ-BR23, and TCM Constitution.

**Table 3:** Schedule of enrolment and assessment

	Baseline	During chemotherapy (every cycle)	Follow-up after chemotherapy (month)				
	0	1–8	6	12	18	24	36
Enrolment							
Informed consent	√						
Eligibility	√						
General assessment							
Age	√						
Sex	√						
Height	√						
Weight	√	√					
Body surface area	√						
Surgical approach	√						
Chemotherapy regimen	√						
Chemotherapy strategy	√						
Pathological stage	√						
Molecular subtype	√						
ECOG PS	√	√	√	√	√	√	√
EORTC QLQ-C30	√	√					
EORTC QLQ-BR23	√	√					
TCM constitution	√	√					
Chest CT	√			√		√	√
*Breast MRI	√			√		√	√
Serum tumour biomarkers	√	√	√	√	√	√	√
Key evaluation							
Blood routine test	√	At least once per week	√	√	√	√	√
Safety examination							
Urine routine test	√	√	√	√	√	√	√
Liver function (ALT, AST, ALP, TBIL, GGT, etc.)	√	√	√	√	√	√	√
Kidney function (CR, BUN, SCR, UA, etc.)	√	√	√	√	√	√	√
Electrocardiogram	√	√	√	√	√	√	√
Adverse events		√	√	√	√	√	√
Data evaluation							
Audit medical records		√	√	√	√	√	√
Ombudsman reviews medical records		√	√	√	√	√	√
Outcome measures		√	√	√	√	√	√

*MRI assessments were conditionally performed according to the patient’s disease status

All results are recorded in the case report form. Participants who show any adverse effects will be reported and followed up by their responsible physicians.

#### Long-term efficacy

1. Three-year, 5-year, and 10-year DFS and OS.
2. Safety evaluation: Blood pressure, heart rate, heart rhythm, respiratory rate, blood routine test, and liver and kidney functions are assessed on a scale of four levels.
  Level 1: Safe—no adverse reactions; no abnormalities in safety indicators.
  Level 2: Comparatively safe—mild adverse reactions are observed; treatment can be continued without any intervention; no abnormality in safety indicators.
  Level 3: Safe issues arise—moderate adverse reactions or mild abnormalities in safety indicators are observed; treatment can be continued with suitable intervention.
  Level 4: Serious adverse reactions—study abortion due to serious adverse reactions; safety indicators are remarkably abnormal.

#### Follow-up schedule

All patients will undergo an 8-week follow-up on blood routine tests, liver and kidney functions, and adverse events. The detailed schedule of enrolment and assessment is listed in Table 2. In consideration of the patient’s safety, outcome assessments are not blinded to the physicians.

### Plans to promote participant retention and complete follow-up {18b}

As mentioned above in {11c}.

### Data management {19}

A data management team consisting of administrative staffs and physicians from the Yueyang Hospital is formed to monitor the study data.

### Confidentiality {27}

The patient’s names are de-identified in the database to protect the patients’ confidentiality.

### Plans for collection, laboratory evaluation, and storage of biological specimens for genetic or molecular analysis in this trial/future use {33}

This trial involves collecting biological specimens for storage. Genetic analysis with the blood samples collected is planned in our ancillary study.

## Statistical methods

### Statistical methods for primary and secondary outcomes {20a}

Data were analysed using SPSS 18.0 (SPSS, lnc., Chicago, IL) and Stata10.0 (StataCorp, College Station, Texas). Multivariate analysis was performed by multiple linear regression, COX regression, or logistic regression, where appropriate. All analyses are based on two-sided test, with *P* values < 0.05 considered as statistically significant.

#### Continuous variables

If the data shows a normal distribution, the data will be expressed as the mean ± standard deviation. Differences between groups are analysed by one-way ANOVA. If the data does not show a normal distribution, the data will be expressed as the median, lower 25% quantile, and upper 75% quantile. Differences between groups are analysed by the Kruskal-Wallis *H* test and the Nemenyi test.

#### Categorical variables

The frequency, relative number, and rate of the data will be calculated. If the data is not in order, the chi-square test or Fisher’s exact test is used to compare the difference between groups. If the data is in order, the rank-sum test is used instead.

#### Survival analysis

The survival curve is plotted by the Kaplan-Meier Method. The log-rank test is used to compare the difference between groups.

### Interim analyses {21b}

No interim analyses are planned in this study.

### Methods for additional analyses (e.g. subgroup analyses) {20b}

None.

### Methods in analysis to handle protocol non-adherence and any statistical methods to handle missing data {20c}

Two datasets will be generated for subsequent analysis: the intent-to-treat (ITT) population which includes all eligible participants and the per-protocol (PP) population which includes only participants who completed the trial.

### Plans to give access to the full protocol, participant-level data, and statistical code {31c}

None.

## Oversight and monitoring

### Composition of the coordinating centre and trial steering committee {5d}

As mentioned above in {19}.

### Composition of the data monitoring committee, its role, and reporting structure {21a}

As mentioned above in {18a}, {19}, and {23}.

### Adverse event reporting and harms {22}

As mentioned above in {11b}.

### Frequency and plans for auditing trial conduct {23}

The frequency and plans for auditing trial conduct are as shown in Table 2. Auditing trial conduct will be performed by a team independent from the investigators and the sponsor.

### Plans for communicating important protocol amendments to relevant parties (e.g. trial participants, ethical committees) {25}

None.

## Dissemination plans {31a}

The final data of the study will be presented at national and international conferences. In addition, a manuscript will be written and submitted to open access peer-reviewed journals.

## Discussion

Due to the huge population size of breast cancer patients in China, the society is now facing a heavy medical burden. Chemotherapy, as one of the routine systemic treatments for breast cancer, inevitably induced CIM which induces toxic side effects and increases the infection risk of patients, thus leading to chemotherapy discontinuation. As a result, the prevention and treatment of CIM is the key to treatment success. Although rhG-CSF is an effective treatment for myelosuppression, it is expensive and has numerous side effects that could impact the patients’ QoL.

In many studies, TCM decoction is found effective to alleviate myelosuppression, which can act as a more economical alternative with fewer side effects and better adherence compared to the existing rhG-CSF treatment. Other than decoctions, moxibustion has also been widely used in preventing and treating diseases. Moxibustion has the function of warming meridians, eliminating phlegm and blood stasis, dispelling Yin and stagnation, replenishing Qi and Yang, rehabilitating and strengthening health, and preventing diseases. It also plays a role in reducing toxicity and improving the efficacy of cancer treatment [32]. Several studies have revealed that moxibustion could effectively prevent chemotherapy-induced leukopenia [33–36]. The clinical characteristics of CIM include symptoms such as pale or yellowing face, dizziness, fatigue, light breath and less talk, palpitation, dull tongue and thin-white tongue coating, and weak pulse, which belong to “consumption” and “blood deficiency” patterns in TCM. The treatment should follow the rule of “treat consumption with warmness” and “treat deficiency with tonification”. These indicated that TCM decoctions and moxibustion may be beneficial to breast cancer patients during chemotherapy.

In order to develop an evidence-based standardized regimen of TCM and moxibustion methodology, conclusive result from a comprehensive scientific study is essential. In this study, moxibustion, or both TCM decoction and moxibustion, is used in combination with rhG-CSF to treat CIM in early-stage breast cancer patients. We intend to create a novel standardized TCM-combined treatment and provide a clinically simple, convenient, and cost-effective intervention that can benefit breast cancer patients’ QoL. Our study integrates TCM and Western medicine to solve unmet medical needs. As a well-organized prospective study, we expect the results could facilitate the translation of TCM concepts into the modern clinical practice of cancer management. This study also aims to standardize a new combination of treatment strategy with integrative Chinese and Western medicine, ultimately forming related expert consensus and clinical guidelines.

## Trial status

The trial started recruitment on 27 December 2017 (Protocol version: v2.1, Date: 28th February 2018). Until 8 December 2019, 218 patients were enrolled. The estimated date of recruitment completion is 31 December 2020.

## Supplementary information

**Additional file 1.** SPIRIT 2013 checklist.

**Additional file 2.** Figure S1. Clinical pathway of each participant

## Data Availability

Not applicable, no datasets are included in this study protocol.

## Abbreviations

ANC: Absolute neutrophil count
AML: Acute myeloid leukaemia
AJCC: American Cancer Joint Committee
CIM: Chemotherapy-induced myelosuppression
EORTC: European Organization for Research and Treatment of Cancer
FN: Febrile neutropenia
MDS: Myelodysplastic syndrome
QoL: Quality-of-life
rhG-CSF: Recombinant human granulocyte colony-stimulating factor
TCM: Traditional Chinese medicine
WBC: White blood cell
