# Supplementary figures and images for: The prophylactic and therapeutic effects of moxibustion combined with traditional Chinese medicine decoction for treating chemotherapy-induced myelosuppression in early-stage breast cancer: study protocol for a randomized controlled trial
Source: Trials. 2020 Oct 12;21:844. doi: 10.1186/s13063-020-04749-6 (PMC7549227; doi:10.1186/s13063-020-04749-6)

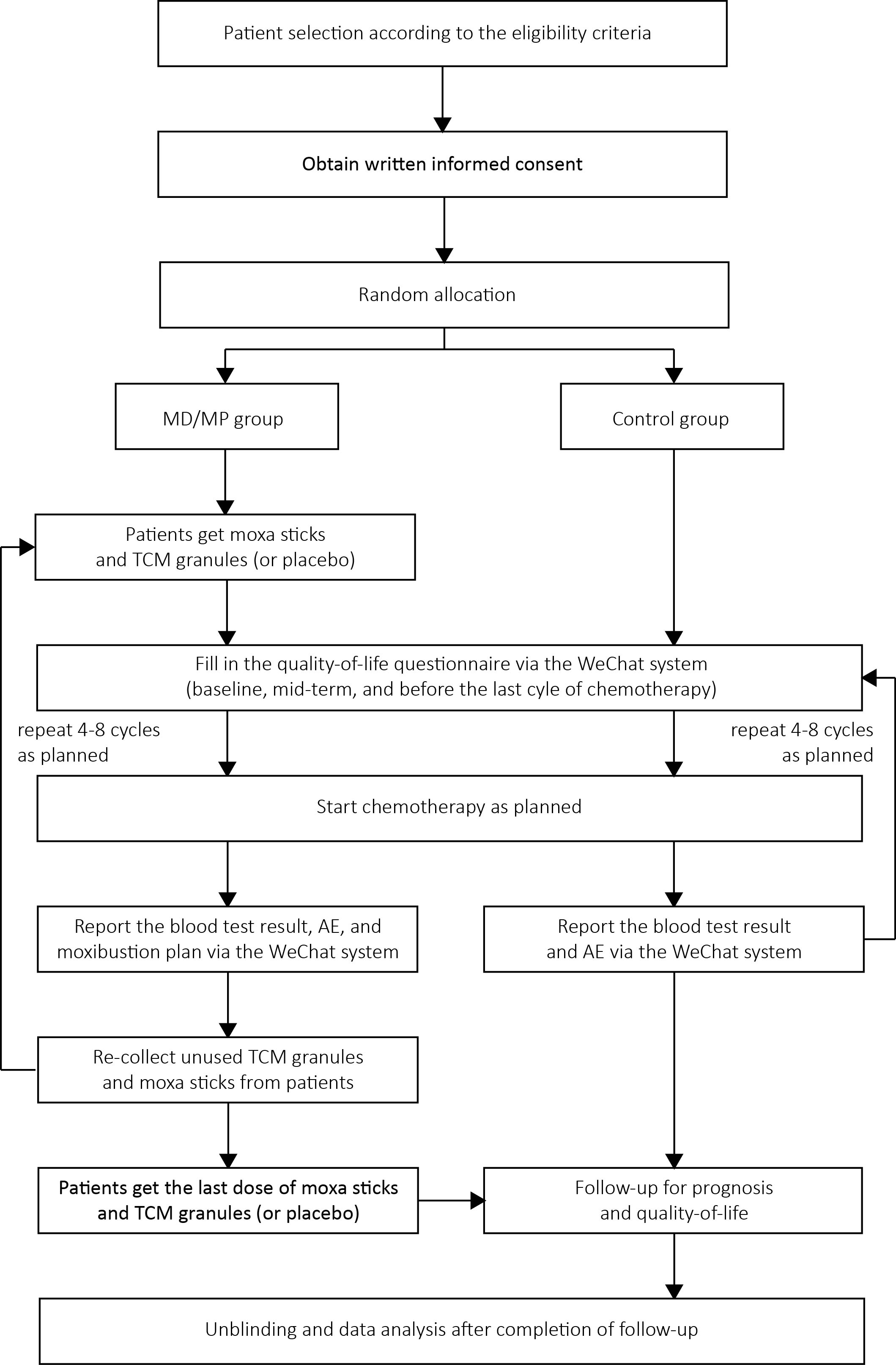

Supplement: Supplementary file 2 — Additional file 2. Figure S1. Clinical pathway of each participant [file 13063_2020_4749_MOESM2_ESM.jpg]
